# Monitoring Scapular Kinematics through Wearable Magneto-Inertial Measurement Units: State of the Art and New Frontiers

**DOI:** 10.3390/s23156940

**Published:** 2023-08-04

**Authors:** Carla Antonacci, Umile Giuseppe Longo, Ara Nazarian, Emiliano Schena, Arianna Carnevale

**Affiliations:** 1 Fondazione Policlinico Universitario Campus Bio-Medico, Via Álvaro del Portillo, 200, 00128 Roma, Italy; carla.antonacci@unicampus.it (C.A.); arianna.carnevale@policlinicocampus.it (A.C.); 2 Unit of Measurements and Biomedical Instrumentation, Università Campus Bio-Medico di Roma, Via Álvaro del Portillo, 21, 00128 Roma, Italy; e.schena@unicampus.it; 3 Research Unit of Orthopaedic and Trauma Surgery, Department of Medicine and Surgery, Università Campus Bio-Medico di Roma, Via Álvaro del Portillo, 21, 00128 Roma, Italy; 4 Carl J. Shapiro Department of Orthopaedic Surgery and Center for Advanced Orthopaedic Studies, Beth Israel Deaconess Medical Center, Harvard Medical School, Boston, MA 20115, USA; anazaria@bidmc.harvard.edu

**Keywords:** scapular kinematics, shoulder, scapula, biomechanics, wearable systems, magneto-inertial measurement units

## Abstract

Monitoring shoulder kinematics, including the scapular segment, is of great relevance in the orthopaedic field. Among wearable systems, magneto-inertial measurement units (M-IMUs) represent a valid alternative for applications in unstructured environments. The aim of this systematic literature review is to report and describe the existing methods to estimate 3D scapular movements through wearable systems integrating M-IMUs. A comprehensive search of PubMed, IEEE Xplore, and Web of Science was performed, and results were included up to May 2023. A total of 14 articles was included. The results showed high heterogeneity among studies regarding calibration procedures, tasks executed, and the population. Two different techniques were described, i.e., with the x-axis aligned with the cranial edge of the scapular spine or positioned on the flat surface of the acromion with the x-axis perpendicular to the scapular spine. Sensor placement affected the scapular motion and, also, the kinematic output. Further studies should be conducted to establish a universal protocol that reduces the variability among studies. Establishing a protocol that can be carried out without difficulty or pain by patients with shoulder musculoskeletal disorders could be of great clinical relevance for patients and clinicians to monitor 3D scapular kinematics in unstructured settings or during common clinical practice.

## 1. Introduction

The shoulder is a complex and kinematically redundant joint: it achieves great mobility through the interplay of three bones (clavicle, humerus, and scapula) and four joints (sternoclavicular, acromioclavicular, glenohumeral, and scapulothoracic) [1,2].

An accurate estimation of shoulder kinematics is an essential aspect in orthopaedic research due to the emerging need to improve clinical diagnostics, evaluate objectively post-surgery functional recovery, and help clinicians to customize the therapy by adapting it to the patient’s specific needs [3,4,5,6,7]. One of the main obstacles in shoulder kinematic analysis is the difficulty of recording and assessing scapular motion non-invasively but accurately [8,9,10,11,12]. The three-dimensional kinematics of the scapulothoracic (ST) joint during dynamic movements can be described by rotations of the scapula on the thorax following the International Society of Biomechanics’ (ISB) recommendations [13]. The scapula rotates in the frontal plane, resulting in a movement called Medio-Lateral Rotation (MLR), posteriorly or anteriorly (tilt) in the sagittal plane, and externally/internally around a longitudinal axis (IER) [2,14,15,16].

From a technological point of view, monitoring scapular motion requires the development of protocols exploiting sensing technology that is, as much as possible, reliable, accurate, and unobtrusive [3,5,17,18,19]. Technologies for monitoring joint kinematics can be classified into two main categories: wearables (WS) and non-wearables (NWS) [17]. WS can be small in size, lightweight, portable, and user-friendly [5,17]. Among WS, Magneto-Inertial Measurement Units (M-IMUs) and textile-based sensors (e.g., flexible conductive wire sensors, flex sensors, and strain sensors) are very suitable for monitoring joint kinematics due to the advantages listed above [17,20,21]. In contrast, NWS are expensive, and their use is restricted to structured laboratory environments, although they are accurate and can perform [5,17,19]. Methods for scapular tracking include the scapular locator or Acromion Marker Cluster (AMC) [22,23,24,25]. A scapular locator is a base with three pins connected to an electromagnetic tracking device, providing scapular orientation in static conditions [22,23,26,27]. An AMC is an L-shaped cluster with three retro-reflective markers [24]. The centre of the AMC is placed on the flat portion of the acromion, with one section pointing anteriorly to the scapular plane and the other following the spine of the scapula for dynamic scapular tracking [24,26,28].

WS overcome the shortcomings of NWS, providing a viable alternative for scapular kinematic monitoring in unstructured environments [5,17,21]. The increasing trend to adopt WS for monitoring 3D scapular kinematics has been promoted by the need to monitor patients during normal Activities of Daily Living (ADLs) and the possibility of recognizing the quality of performed physical exercises and preventing movement disorders [16,17,21,29]. In addition, alternative solutions to analyse and identify the principal shoulder movements are based on multi-sensor approaches [30,31,32,33,34]. For example, electromyographic sensors (EMGs) and accelerometers were incorporated together to detect anatomical contractions and functional skeletal movements of the shoulder [33,34].

Moreover, among WS, M-IMUs are commonly used for tracking scapular kinematics [17,35,36,37,38]. M-IMUs incorporate 3D accelerometers, gyroscopes, and magnetometers to provide 3D orientation [3,17,39]. Despite the existing literature on scapular tracking using wearable M-IMUs, a systematic review, pooling existing knowledge so that a general consensus can be reached, is lacking.

The aim of this systematic literature review is to report and describe the existing methods to estimate 3D scapular movements through wearable systems integrating M-IMUs. Based on this review, recommendations for scapular motion tracking through M-IMUs and future research are formulated.

## 2. Methods

### 2.1. Literature Search Strategy and Eligibility Criteria

This systematic review was developed following PRISMA (Preferred Reporting Items for Systematic Reviews and Meta-Analyses) guidelines [40]. Articles were selected from three different databases, namely, PubMed, IEEE Xplore, and Web of Science. Free text terms and Mesh (Medical Subject Headings) were combined using logical Boolean operators (OR, AND). In each database, the search was executed as follows: (“shoulder kinematics” OR “shoulder joint” OR “shoulder biomechanics” OR “scapular biomechanics” OR “scapulothoracic joint” OR “scapular kinematics”) AND (wearable* OR “wearable system*” OR “wearable device*” OR “wearable technolog*” OR “wearable sensor system*” OR “magneto-inertial sensor*” OR “magneto-inertial measurement unit*” OR “M-IMU*” OR “inertial and magnetic sensor*” OR “inertial-magnetic measurement unit*”). All studies published up to May 2023 were considered. The title and abstract of articles retrieved from the search were independently screened by two reviewers (C.A. and U.G.L.). In cases of disagreement, the final consensus for inclusion/exclusion was reached after discussion with two other reviewers (E.S. and A.C.). Articles were screened following the established inclusion/exclusion criteria.

**Inclusion criteria.** This systematic review includes studies written in the English language that met the following criteria:

1. Papers are published in a journal or presented at a conference.
2. The studies used wearable M-IMUs to track scapular kinematics.
3. Sensors are placed directly on the human skin via an adhesive, embedded within pockets, straps, or integrated into fabrics.
4. Upper limb functional tasks are investigated.

**Exclusion criteria.** Articles were excluded if at least one of the following criteria was met:

1. Reviews, books.
2. Use of exoskeleton or robotic systems.
3. Scapular kinematics is not included in the upper limb motion analysis.
4. Wearable devices are not directly tested on humans.

### 2.2. Data Collection Process

Data collection was executed on all articles in the listed databases satisfying the inclusion criteria. Data were collected based on the following checklist: first author and publication year; brand, number, and placement of the M-IMUs; system used as the gold standard to evaluate the wearable device’s performance; calibration method; characteristics of the subjects included in the study (e.g., presence of pathologies, sex, age, height, weight, and BMI); tasks executed; the aim of the study; systems’ performance regarding scapular kinematics parameters.

## 3. Results

**Figure 1:**
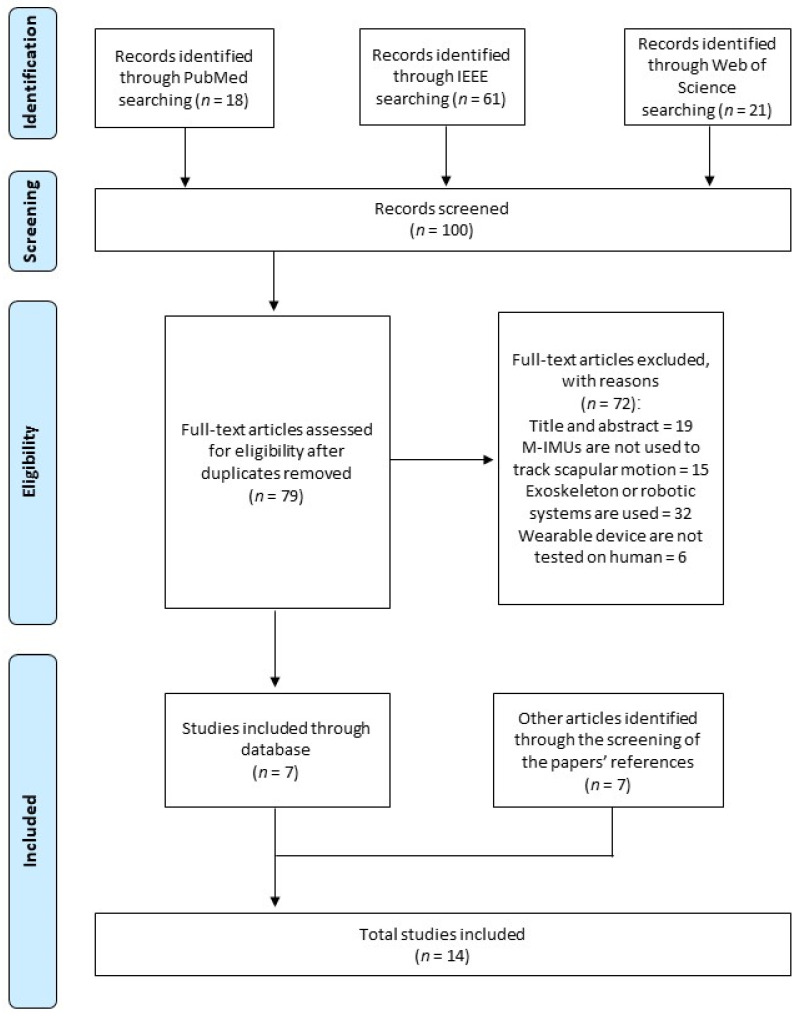
PRISMA 2009 flow diagram.

A total of 100 papers was found by searching the listed databases. Screening of the selected articles’ references identified seven other papers. A total of 14 articles was included in this systematic review (Figure 1). Details of the selected papers are summarized in Table 1 and Table 2. Only studies reporting quantitative results on scapular kinematics are included in Table 2 [36,37,38,41,42,43,44,45,46,47,48].

### 3.1. Sensors Positioning

Of the included studies, ten articles used from three to five M-IMUs for unilateral upper limb monitoring [36,41,42,43,44,45,46,49,50], while only four authors used from five to seven for bilateral monitoring [37,38,47,48].

**Figure 2:**
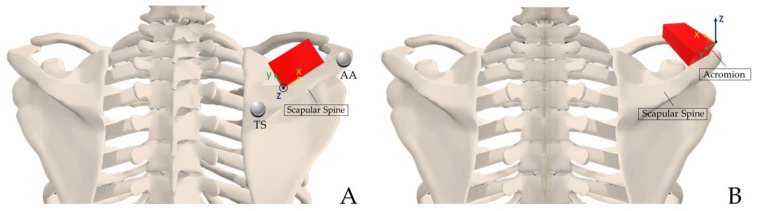
Scapular M-IMU (in red) positioning technique (unilateral case): M-IMU was placed over the central third between the angulus acromialis (AA) and the trigonum spinae (TS) aligning the x-axis of the sensor with the cranial edge of the scapular spine (**A**); M-IMU was positioned on the flat surface of the acromion with the x-axis perpendicular to the scapular spine (**B**).

In the selected articles, the upper arm sensor was placed on the posterior and distal end of the humerus, positioned halfway between the medial and lateral epicondyle [14,17,18,19,21,22,23,24,25,26,27,28,29]. The forearm sensor was used only in a few articles, and it was placed dorsally at the distal end close to the ulnar process [18,19,23,27,28,29]. Moreover, in the study of Höglund et al., two different positioning techniques to track upper limb motion were evaluated and compared: a total of four M-IMUs were placed centred on the lateral and dorsal distal end of both the upper arm and forearm [20]. In all the reviewed articles, the thorax sensor was placed on the chest under the jugular notch [35,36,37,38,42,43,44,45,46,47,48,49,50]. Its role was crucial because data from scapular M-IMUs are used for tracking rotational and translational movements of the scapula with respect to the thorax M-IMUs [17,51]. In the literature, two different positioning techniques were described for scapular M-IMUs (Figure 2). The most common M-IMU positioning to track 3D scapular kinematics was to align the x-axis of the sensor with the cranial edge of the scapular spine [35,36,38,42,43,44,45,46,47,48,49]. In particular, the scapula M-IMU was placed over the central third between the angulus acromialis (AA) and the trigonum spinae (TS) by palpating the scapular spine from the most lateral part of the acromion to the most medial aspect of the scapula [35,36,38,42,43,44,45,46,47,49]. In the other positioning technique, the M-IMU for evaluating scapular rotation was positioned on the flat surface of the acromion with the x-axis of the sensor perpendicular to the scapular spine [37,50].

### 3.2. Gold Standard

In six studies, the performance evaluation of wearable systems based on M-IMUs for monitoring scapular kinematics was compared with optoelectronic motion capture systems (MOCAPs) as the gold standard [35,36,37,38,41,50].

In the study of Cutti et al., M-IMU sensors positioned on the thorax, upper arm, and forearm were mounted on rigid clusters of four markers [35]. In order to monitor 3D scapular kinematics, a specific and lightweight cluster was fabricated. The clusters were then attached directly to the subjects’ skin to track upper limb movements with both MOCAPs and wearable M-IMUs [35]. This solution ensured that the same negligible soft-tissue artifacts affected both tracking devices [35]. Following this experimental protocol, they obtained a Root Mean Square Error (RMSE) between 0.2° and 3.2° for 97% of the angles data (i.e., elbow, humerothoracic, and scapulothoracic joint angles). These results showed wearable M-IMUs’ ability to provide angles data highly correlated to the MOCAPs during the execution of bilateral upper arm movements [35]. Similarly, Grip et al. positioned M-IMUs on clusters showing high validity for scapular segments when compared to MOCAPs (−0.2 ± 1.2°) [38].

Other studies placed sensors directly on the skin, and they demonstrated that angles’ trajectories were relatively similar between M-IMUs and MOCAPs [36,37,41,50]. However, there was a systematic error between the scapular kinematics reported from both motion capture systems [37,50]. This constant scapular angle offset was likely responsible for some of the relatively higher RMSE values (RMSE = 25.6° for scapular IER during overhead lift) [37].

### 3.3. Calibration

Sensor-to-segment calibration is an essential procedure for defining a relationship between human body segments and M-IMUs [5,17,21]. For scapular monitoring, single or double and static or dynamic calibrations were proposed and investigated [52,53]. Static calibration consisted of strictly aligning the axes of the sensors with those of the body segments [18]. During a dynamic calibration procedure, the subject performed a series of specific functional movements such as elbow flexion–extension, forearm pronation–supination, shoulder flexion–extension, and abduction–adduction [18,34]. In addition, the association between static and dynamic calibration, known as double calibration, was used [52,53].

The single calibration was the most used [35,36,38,42,44,46,47,50]. In several of the reviewed papers, the subject was instructed to stand still for a few seconds, with their shoulders and trunk in the neutral position, with both arms alongside the body, perpendicular to the ground, and with the thumb pointing forward [35,38,50]. In other studies, this static measure was performed with the subject standing in an upright position, the upper arm along the trunk for neutral humerus internal/external rotation, and the elbow flexed at 90° [36,42,44,46,47]. A double static calibration procedure was performed by recording the scapular orientation in a neutral position and at maximum humeral elevation in both the sagittal and scapular planes [37,47].

A comparison of different calibration techniques was performed in one study [44]. Static posture with the trunk upright, the upper arm along the trunk, and the elbow in a 90° flexion and double dynamic calibration were compared by van den Noort et al. [44]. The latter technique consisted of several static measurements performed at 0°, 30°, 60°, 90°, and 120° of humerothoracic elevation [52]. Moreover, in the double calibration procedure, an active humeral anteflexion from 0° to 120° in the sagittal plane and humeral abduction from 0° to 120° in the frontal plane were recorded [52]. In conclusion, the authors suggested double calibration, especially in subjects with a high body mass index, to prevent the underestimation of scapular MLR [44].

### 3.4. Tasks Executed

Following sensor placement and completing the sensor-to-segment calibration, all the experimental protocols of the reviewed articles included the execution of several static positions or dynamic movements. The tasks most used for the estimation of three-dimensional ST joint angles were flexion in the sagittal plane, abduction in the frontal plane, and elevation of the upper limb in the scapular plane [35,36,37,38,43,44,45,46,47,48,49]. Some studies assessed shoulder internal/external rotation [35,38,46,48]. Tasks relating to ADLs, such as hand to mouth, hand to neck, or hand to back, were also evaluated [35,38]. The experimental protocol of Friesen et al. included eight tasks of the WRAFT protocol [37]. The three-dimensional ST joint angles were extracted during the execution of the elevation tasks (e.g., flexion in the sagittal plane, abduction in the frontal plane, comb hair, overhead reach, and overhead lift) [37]. Höglund et al.’s study evaluated subjects while performing nine standardized arm movements included in the modified Mallet scale [50]. During elevation movements (such as shoulder flexion–extension and abduction–adduction), only the scapular MLR and tilt were evaluated [50]. Scapular IER was assessed during the execution of tasks simulating ADLs [50].

**Table 1:** Three-dimensional scapular motion monitoring with wearable M-IMUs for application in healthy subjects and patients.

First Author, Year	M-IMUs: Brand Numbers and Placements	Gold Standard	Calibration Method	Participants	Tasks Executed	Aim
Cutti et al., 2008 [35]	MT9B (Xsens Technologies, NL) Unilateral (*n* = 3): thorax, scapulae (aligning with the scapular spine), and humerus	MOCAP (Vicon 460, Oxford Metrics, UK)	SC: the subject is instructed to stand still, with his back straight and with both arms alongside the body, perpendicular to the ground for 10 s	HS (*n* = 1) 1M 23.3 Y	Elbow FE, elbow PS, shoulder FE, IR, and ER shoulder-girdle elevation depression, PR, shoulder IR and ER with the arm abducted 90°, a shoulder AB-AD in the frontal plane, hand-to-nape task in the sagittal plane, and a hand-to-top-of-head task in the frontal plane	Develop a protocol to measure ST, HT joint angles, and elbow kinematics in ambulatory settings using M-IMUs
Parel et al., 2012 [36]	MTx sensor units (Xsens Technologies, NL) Unilateral (*n* = 3): thorax, scapulae (aligning with the scapular spine), and humerus	-	SC (static posture): upright position, elbow flexed at 90°, neutral forearm rotation, humerus perpendicular to the ground and in neutral rotation	P with MSDs (*n* = 20) 8F, 12M 28.3 ± 5.5 Y BMI 22.4 ± 1.8 HS (*n* = 20) 7F, 13M 43.9 ± 19.9 Y BMI 23.9 ± 4.8	Humeral elevation in the sagittal (FE) and scapular (AB-AD) plane	Intra- and inter-operator agreement of ISEO protocol (INAIL Shoulder and Elbow Outpatient protocol based on inertial and magnetic sensors).
Parel et al., 2014 [41]	MTx sensor units (Xsens Technologies, NL) Unilateral (*n* = 3): thorax, scapulae (aligning with the scapular spine), and humerus	MOCAP (Motion Analysis Corporation; Santa Rosa, CA USA)	SC (static posture): upright position, elbow flexed at 90°, neutral forearm rotation, humerus perpendicular to the ground and in neutral rotation	HS (*n* = 23) 10F, 13M 29 ± 8 Y	Humeral elevation in the sagittal (FE) and scapular (AB-AD) plane	Comparison of two shoulder kinematic protocols.
van den Noort et al., 2014 [42]	MTw wireless sensor units (Xsens Technologies, NL) Unilateral (*n* = 4): thorax, scapulae (aligning with the scapular spine), upper arm, and lower arm	-	SC (static posture for few seconds): trunk upright, the upper arm along the trunk for neutral humerus internal/external rotation, and the elbow in 90° flexion	HS (*n* = 20) 17F, 3M 36 ± 11 Y BMI: 22 ± 2 Physical therapists (*n* = 2)	Elbow FE and shoulder PS	Intra- and inter-operator reliability and precision of the scapular kinematics using M-IMU.
Roldán-Jiménez et al., 2015 [43]	InertiaCube3™ (Intersense Inc., Billerica, MA, USA) Unilateral (*n* = 4): thorax, scapulae (along the scapular spine), humerus, and distal surface of the ulna and radius	-	-	Young HS (*n* = 11) 3F, 8M	Subject performed 180° right shoulder AB-AD and 180° right shoulder FE with the elbow extended, the wrist in neutral position, and the palmar area of the hand toward the midline at the beginning and end of the movement	Analyse upper-limb motions in the three anatomical axes.
van den Noort et al., 2015 [44]	MTw wireless sensor units (Xsens Technologies, NL) Unilateral (*n* = 4): thorax, scapulae (aligning with the scapular spine), upper arm, and lower arm	-	SC (static posture with trunk upright, upper arm along the trunk, elbow in 90° flexion, elbow FE and PS); DC (measurements were performed at 0° HT elevation, and at 30°, 60°, 90°, and 120° of active static HT elevation with elbow fully extended and thumb pointing lateral or up)	P with scapular dyskinesis (*n* = 10)	Bilateral active FE in the sagittal plane, bilateral, active AB-AD in the frontal plane (elbow fully extended and thumb pointing up)	Evaluate the change in 3D scapular kinematics caused by SC and DC with a scapular locator versus ISEO-protocol; assess the difference in 3D scapular kinematics between static posture and dynamic humeral elevation.
Roldán Jiménez et al., 2016 [45]	InertiaCube3™ (Intersense Inc., Billerica, MA, USA) Unilateral (*n* = 4): thorax, scapulae (along the scapular spine), humerus, and distal surface of the ulna and radius	-	-	Young HS (*n* = 11) 8F, 3M Older HS (*n* = 14) 9F, 5M	Shoulder abduction in the coronal plane and shoulder flexion in the sagittal plane	Analyse age-related differences in shoulder kinematics between young and older asymptomatic adults.
Carbonaro et al., 2018 [49]	MTw wireless sensor units (Xsens Technologies, NL) Unilateral (*n* = 3): thorax, scapulae (along the scapular spine), and humerus	-	-	Physiotherapists (*n* = 2) HS (*n* = 5)	ER arm AB-AD	Define a new set of WS capable of evaluating the shoulder angles to characterize classic shoulder rehabilitation tasks and discriminate correct and incorrect movements.
Ajčević et al., 2020 [46]	MTw wireless sensor units (Xsens Technologies, NL) Unilateral (*n* = 3): thorax, scapulae (along the scapular spine), and humerus	-	SC: upright position, elbow flexed at 90°	P with AC (*n* = 6) 3F, 3M 53.8 ± 4.3 Y HS (*n* = 7) 3F, 4M 41.3 ± 4.3 Y	Micro-mobilization of accessory clavicula, AC and SCl joints, scapula, cervical and dorsal rachis. Dynamic mobilizations: anterior flexion, abduction, ER, and IR and postural active exercises	Investigate the possibility to quantitatively evaluate patients who suffer from capsulate-related deficit versus healthy controls and assess treatment efficacy.
Iban et al., 2020 [47]	Bilateral (*n* = 5): one at the manubrium sterni, two on each suprascapular fossae and two over the lateral aspect of both arms	-	SC: subject standing upright, the humerus positioned alongside the body and the elbow flexed at 90°	HS (*n* = 25) 12F, 13M 37 ± 11.1 Y	FE and AB-AD movements	Evaluate the intra- and interobserver reproducibility for assessing the 3D shoulder kinematics in an outpatient setting.
Höglund et al., 2021 [50]	Unilateral (*n* = 7): one sensor on thorax, two on the scapula (the first on the flat surface of acromion and the second aligned with the scapular spine), two on the upper arm, and two on the forearm	MOCAP	SC: the arms hanging vertically, alongside the participant, with the palm of the hand pointing medially	HS (*n* = 11) 5F, 6M 28 ± 6.5 Y	Nine arm-movement tasks based on the Modified Mallet Scale [54]	Evaluate how sensor placement affects kinematic outputs in the assessment of motion of the arm, shoulder, and scapula.
Grip et al., 2022 [38]	Bilateral (*n* = 7): thorax, scapula sensors (cranially on the middle part of spina scapulae), upper arm, and forearm sensors	MOCAP (Oqus, Qualisys AB, Gothenburg, Sweden)	SC: the arms alongside the body with palms facing the body	BPBI group (*n* = 6) 8–22 Y 4F, 2M Control group (*n* = 9) 7–25 Y 6F, 3M	Shoulder FE in the sagittal plane, elbow FE in the sagittal plane, forearm PS, maximal AB-AD, ER, IR, hand to neck, hand to spine, and hand to mouth	Evaluate the validity of a wearable M-IMUs-based system in healthy individuals; assess the test–retest and inter-rater reliability in a group of BPBI patients and non-asymptomatic individuals.
Friesen et al., 2023 [37]	XSens Awinda (Xsens Technologies, NL) Bilateral (*n* = 5): sternum, bilateral posterior, and distal end of the humeri on scapulae (with the x-axis of the sensor perpendicular to the scapular spine or aligned with mid-scapular spine)	MOCAP (Vicon, Oxford, UK)	DC: at neutral position and at maximum humeral elevation	HS (*n* = 30) 15F, 15M 24 ± 4 Y Height 1.7 ± 0.1 m Weight 78.6 ± 16.9 kg	AB-AD in the frontal plane, FE in the sagittal plane, and eight tasks of the WRAFT protocol [55]	Assess the reliability of scapular motion M-IMU measurements compared to the gold standard; compare scapular M-IMU placement to assess which location (acromion or spine) was the best for the validity and reliability of scapular kinematics.
Reina et al., 2023 [48]	ShowMotion (NCS Lab srl, Modena, Italy) Bilateral (*n* = 7): thorax, scapula sensors (on suprascapular fossae), upper arm, and forearm sensors	-	-	P with RTSA (*n* = 14) 7F, 7M	FE, AB-AD in the scapular plane, IR/ER with elbow abducted to the thorax, and IR/ER with shoulder abduction at 90° and elbow flexed to 90°	Assess upper extremity kinematics and active ROM in patients who underwent RTSA compared with the contralateral side and quantify the ST motion.

HS—Healthy Subjects, MSDs—Musculoskeletal Disorders, AC—Adhesive Capsulitis, P—Patient, Y—Year, M—Male, F—Female, SC—Single Calibration, DC—Double Calibration, AC—Acromioclavicular joint, SCl—Sternoclavicular joint, ER—External Rotation, IR—Internal Rotation, FE—Flexion–Extension, AB-AD—Abduction–Adductions, AMC—Acromion Marker Clusters, WS—Wearable Sensor, HT—humerothoracic, PS—Prono-supination, PR—Prono-retraction, ME—Medial Epicondyles, LE—Lateral Epicondyles, MOCAP—Motion Optical Capture System, GH—Glenohumeral, ST—Scapulothoracic, RTSA—Reverse Total Shoulder Artroplasty, BPBI—Brachial Plexus Birth Injury., ‘-’—data not available.

**Table 2:** Scapular parameters and performance coefficients evaluated by the selected papers.

Study, Year	Tasks Executed	Scapular Parameters and Performance Coefficients		
		Tilt	MLR	IER
Parel et al., 2012 [36]	FE Ab-Ad	CMC (SD) = 0.95° (0.05°), SEM = 3.1°, SDD = 8.5° CMC (SD) = 0.94° (0.06°), SEM = 2.7°, SDD = 7.4°	CMC (SD) = 0.96° (0.04°), SEM = 2.2°, SDD = 6.2° CMC (SD) = 0.95° (0.06°), SEM = 1.8°, SDD = 4.9°	CMC (SD) = 0.85° (0.11°), SEM = 2.6°, SDD = 7.1° CMC (SD) = 0.87° (0.11°), SEM = 3.0°, SDD = 8.3°
Parel et al., 2014 [41]	FE (max HT) Ab-Ad (max HT)	SEM = 1.7°, RMSE = 1.5° SEM = 2.2°, RMSE = 2.15°	SEM = 2.6°, RMSE = 2.75° SEM = 3.3°, RMSE = 3.42°	SEM = 2.2°, RMSE = 1.96° SEM = 2.2°, RMSE = 2.3°
van den Noort et al., 2014 [42]	Flexion (max HT) Abduction (max HT)	ICC = 0.67, SEM = 5, SDD = 13 ICC = 0.71, SEM = 5, SDD = 13	ICC = 0.88, SEM = 3, SDD = 9 ICC = 0.84, SEM = 4, SDD = 10	ICC = 0.80, SEM = 5, SDD = 14 ICC = 0.78, SEM = 5, SDD = 14
Roldán Jiménez et al., 2015 [43]	Flexion Abduction	Mean ROM (SD) = 4.1° (16.9°) Mean ROM (SD) = −5.5° (12.3°)	Mean ROM (SD) = −7.7° (48.6°) Mean ROM (SD) = −5.9° (9.5°)	Mean ROM (SD) = 37.8° (6.3°) Mean ROM (SD) = 36.6° (10.2°)
van den Noort et al., 2015 [44]	Humeral abduction (max = 150°)	Mean difference = −8.4° Standard Error = 8.8°	Mean difference = 14.4° Standard Error = 10.1°	Mean difference = −12.1° Standard Error = 24.8°
Roldán-Jiménez et al., 2016 [45]	FE (young group) FE (older group) Ab-Ad (young group) Ab-Ad (older group)	Mean ROM = 17.8° (8.9°–26.7°) Mean ROM = 23.2° (18.7°–27.7°) Mean ROM = 10.7° (5.6°–15.8°) Mean ROM = 15.6° (11.2°–19.9°)	Mean ROM = 19° (9.3°–28.6°) Mean ROM = 5.4° (3.5°–7.4°) Mean ROM = 10.1° (6.14°–14.2°) Mean ROM = 5.73° (2.52°–8.95°)	Mean ROM = 44° (39.2°–48.8°) Mean ROM = 29° (25°–33.1°) Mean ROM = 42.1° (36.3°–47.9°) Mean ROM = 33.8° (27.5°–40.1°)
Ajčević et al., 2020 [46]	Ab-Ad (pre-treatment) Ab-Ad (post-treatments)	Mean ROM (SD) = 21° ± 7.1° Mean ROM (SD) = 34.6° ± 7.7°		
Iban et al., 2020 [47]	Flexion (max HT) Abduction (max HT)	Mean ROM (SD) = 12.9° (6.65°) Mean ROM (SD) = 10.3° (6.1°)	Mean ROM (SD) = 25.2° (5.44°) Mean ROM (SD) = 24.0° (5.29°)	Mean ROM (SD) = −4.57° (5.20°) Mean ROM (SD) = −3.53° (5.66°)
Grip et al., 2022 [38]	FE Ab-Ad ER Hand-to-mouth IR	ICC = 0.92 ICC = 0.88 ICC = 0.92 ICC = 0.93 ICC = 0.97	ICC = 0.71 ICC = 0.80 ICC = 0.94 ICC = 0.84 ICC = 0.80	ICC = 0.71 ICC = 0.71 ICC = 0.88 ICC = 0.85 ICC = 0.92
Friesen et al., 2023 [37] *	Combining Hair (Acromion) Combining Hair (Spine) Overhead Reach (Acromion) Overhead Reach (Spine) Overhead Lift (Acromion) Overhead Lift (Spine) Abduction (Acromion) Abduction (Spine) Flexion (Acromion) Flexion (Spine)	ICC = 0.504, RMSE = 14.7° ICC = −0.029, RMSE = 15.8° ICC = 0.209, RMSE = 14.1° ICC = −0.478, RMSE = 24.5° ICC = 0.267, RMSE = 14.7° ICC = −0.611, RMSE = 27.7° ICC = 0.426, RMSE = 15.0° ICC = 0.180, RMSE = 24.7° ICC = 0.446, RMSE = 18.8° ICC = −0.006, RMSE = 23.0°	ICC = 0.740, RMSE = 7.0° ICC = 0.606, RMSE = 11.3° ICC = 0.504, RMSE = 11.8° ICC = 0.720, RMSE = 8.8° ICC = 0.638, RMSE = 12.8° ICC = 0.523, RMSE = 12.1° ICC = 0.646, RMSE = 9.8° ICC = 0.652, RMSE = 10.6° ICC = 0.664, RMSE = 9.4° ICC = 0.312, RMSE = 12.7°	ICC = 0.855, RMSE = 9.9° ICC = 0.651, RMSE = 10.9° ICC = 0.677, RMSE = 13.4° ICC = 0.22, RMSE = 15.0° ICC = 0.670, RMSE = 17.9° ICC = −1.069, RMSE = 25.6° ICC = 0.849, RMSE = 12.2° ICC = 0.207, RMSE = 20.8° ICC = 0.914, RMSE = 10.8° ICC = 0.433, RMSE = 15.9°
Reina et al., 2023 [48]	FE (path. − max HT) FE (healthy − max HT) Ab-Ad (path. − max HT) Ab-Ad (healthy − max HT)	Mean ROM (SD) = 28.9° (7.5°) Mean ROM (SD) = 22.0° (8.9°) Mean ROM (SD) = 20.3° (6.7°) Mean ROM (SD) = 19.0° (6.1°)	Mean ROM (SD) = 34.1° (9.9°) Mean ROM (SD) = 31.4° (13.0°) Mean ROM (SD) = 27.10° (6.7) Mean ROM (SD) = 23.8° (5.6)	Mean ROM (SD) = −12.7° (9.0°) Mean ROM (SD) = −8.7° (8.6°) Mean ROM (SD) = −12.9° (7.8°) Mean ROM (SD) = −12.4° (6.8°)

* Evaluated at the max degree of humeral elevation. MLR—Medio-Lateral Rotations, IER—Internal and External Rotation, FE—Flexion–Extension, AB-AD—Abduction–Adduction, HT—Humerothoracic, RMSE—Root Mean Square Error, SD—Standard Deviation, ICC—Intra-Class Coefficient, CMC—Coefficient of Multiple Correlation, SEM—Standard Error of Measurement, ROM—Range of Motion.

### 3.5. Scapular Kinematics and Systems’ Performance

The reviewed studies evaluated the performance of the proposed wearable systems based on M-IMUs, such as their validity or reliability, or compared scapular kinematics in the healthy individuals’ and patients’ populations [35,36,37,38,41,42,43,44,45,46,47,48,50].

Studies comparing the M-IMU-based wearable systems with MOCAPs obtained good to excellent results [35,36,37,38,41,50], although the results worsen as the complexity of the tasks increases [50]. In fact, for example, Grip et al.’s study demonstrated that, if comparing their results extracted by wearable M-IMU-based systems with the gold standard, the ICC values were close to 1 so they were high in reliability [38]. Similarly, in Friesen’s study, an RMSE of 25.6° and of 20.8° in scapular IER during the execution of an overhead lift and abduction, respectively, were recorded with an M-IMU placed on the acromion [37]. In addition, this study aimed to assess which location (acromion or spine) was the best for the reliability of scapular motion. They found that the best positioning technique is to place the M-IMU on the acromion especially for IER across most tasks (minimum ICC recorded during overhead lift was of 0.670 compared to the corresponding value of ICC = −1.069).

The mean difference and standard error were evaluated by van den Noort et al., comparing two different calibration techniques [25]. The results showed high differences in the ROM and standard deviation between single and double calibrations (mean differences of −8.4°, 14.4°, and −12.1° were recorded between scapular tilt, MLR, and IER) [25].

Other studies compare the scapular measurements recorded by two different operators (inter-reliability) [36,42]. The results were excellent especially for scapular IER (ICC = 0.80 during flexion and ICC = 0.78 during abduction) and MLR (ICC = 0.88 during flexion and ICC = 0.84 during abduction) in van den Noort et al.’s paper and for scapular tilt, MLR, and IER (CMC > 0.90°) during the execution of flexion and abduction in Parel’s study [36,42].

Some articles aimed to compare the performance of different populations included in their studies [45]. Roldán-Jiménez’s study investigated the differences in scapular three-dimensional motions between young and older asymptomatic adults [45]. They found that subjects presented less scapular MLR and IER values when their age increases [45]. Other authors aimed to analyse the performance of patients [46,48]. For example, Reina et al. included a group of subjects undergoing reverse total shoulder arthroplasty (RTSA), and their work aimed to compare the pathological upper limb with the contralateral side [48]. The RTSA scapular results had high standard deviations (SD): in fact, SD values of 20.3° and 6.1° were recorded in scapular tilts during abduction in the pathological and healthy side, respectively [48]. Similarly, Ajčević et al. enrolled subjects with adhesive capsulitis [46]. They found a significant increase of the scapular ROM (mean ROM of 21° and 34.6° were extracted before vs. after rehabilitation treatments) [46].

## 4. Discussion

Advances in wearable systems based on M-IMUs have shown promising results in recording three-dimensional scapular motions. However, the lack of standardized protocols, sensor-to-segment calibration, and M-IMU positioning makes it difficult to identify a universal procedure that reduces the variability among studies.

Defining the best sensor positioning for scapular kinematics monitoring through wearable M-IMUs is crucial for providing clinically relevant information [42,50,56]. A protocol called ISEO (INAIL Shoulder and Elbow Outpatient protocol) has been developed and tested for estimate scapular kinematics through M-IMUs, showing a similar within-protocol repeatability for the protocol that uses a scapula tracker (RMSE = 2.35°, SEM = 2.5°) and the ISEO protocol (RMSE = 2.24°, SEM = 2.3°) [35,41]. Höglund et al. demonstrated that positioning the sensor cranially along the scapular spine reduces the influence of underlying muscle and skin movements [50]. Despite this, Friesen et al. demonstrated that placing the scapular M-IMU on the acromion corresponds to a higher reliability than with spine placement [37].

### 4.1. Application in a Clinical Scenario

Patients suffering from shoulder joint dysfunctions may experience shoulder pain and restrictions in performing ADLs [57,58,59]. Due to difficulty performing free-pain movements, they often develop compensatory scapular movements to maintain a physiologic range of motion [60]. The quantitative evaluation of scapular kinematics with wearable M-IMUs in clinical scenarios represents a valid solution that complements subjective assessments using clinical scales [61,62,63,64].

Among the reviewed studies, some applied wearable systems integrating M-IMUs for monitoring scapular kinematics in children with brachial plexus birth injury, patients suffering from scapular dyskinesis, or patients undergoing reverse total shoulder prosthesis [38,44,48]. A bilateral configuration of wearable M-IMUs was used for evaluating 3D scapular rotations in 14 patients after primary reverse total shoulder arthroplasty [48]. The results showed a greater contribution of scapular movement in pathological shoulders than in the contralateral healthy shoulder (pathological vs. healthy side at 90°–120° of flexion (mean ± std): tilt, 28.9° ± 7.5° vs. 22.0° ± 8.9°; MLR, 34.1° ± 9.9° vs. 31.4° ± 13.0°; IER, −12.7° ± 9.0° vs. −8.7° ± 8.6°) [48]. Similarly, for elevations in the frontal plane, these results showed how a greater ST contribution is essential to ensure the same level of humeral elevation [48]. In another study, patients with scapular dyskinesis were invited to perform elevations in the sagittal and frontal planes while the M-IMUs measured 3D scapular kinematics to investigate the effect of different calibration procedures on measurements [44]. Although the results showed that scapular locator calibration is necessary when using M-IMUs for scapular monitoring and that for elevations above 90°, double calibration avoids the underestimation of scapular MLR, further investigation would be useful in more homogeneous populations to confirm the validity of the proposed method [44]. The validity of scapular motion monitoring was high when compared to MOCAPs (−0.2 ± 1.2°) during hand to neck, hand to spine, hand to mouth, and internal rotations performed by patients with brachial plexus birth injury, but the results should be cautiously interpreted given the undesirable contribution of underlying soft tissues [38].

### 4.2. Recommendations and New Frontiers

Placing the scapular M-IMU with the x-axis aligned with the upper edge of the scapular spine over the central third between the angulus acromialis and the trigonum spinae would seem appropriate. Firstly, as some authors suggest, positioning the scapular M-IMUs aligned the x-axis of the sensor with the upper edge of the scapular spine allowed for the defining of the scapular sensor units’ system of reference axes as close as possible to ISB recommendations [13,35,36]. The proposed sequence was consistent with both research and clinical-based representations of scapular motions [13]. The three-dimensional scapulothoracic kinematics are then described by three independent angles obtained with the sequences of Euler angles: changing the sequence resulted in significant alterations in the description of motion [13,35,36]. Secondly, the positioning of the scapular M-IMU along the scapular spine was the simplest techniques of the methods described in the reviewed articles [50]. Simple yet reliable protocols and procedures should be further investigated to use wearable systems integrating M-IMUs to monitor scapular kinematics in patients with shoulder musculoskeletal disorders. The single calibration approach with the subject standing with a straight back and both arms along the body, perpendicular to the ground, could also be executed by patients unable to perform complete upper-limb movements or assuming a static position with the elbow at 90° for several seconds because of pathological conditions. From this point of view, simplified segment-to-sensor calibration methods and sensor placements might allow for the easy monitoring of scapular kinematics during rehabilitation.

However, further studies should be conducted to establish a validated and universal protocol (including standardized M-IMU placement and calibration procedures). Establishing a protocol that can be carried out without difficulty or pain for patients with shoulder musculoskeletal disorders could be of great clinical relevance for patients and clinicians to monitor 3D scapular kinematics in unstructured settings or during common clinical practice, respectively.

## 5. Conclusions

Wearable systems equipped with M-IMUs are becoming a promising tool for evaluating the 3D scapular motion in orthopaedic clinical research. However, evaluating scapular kinematics by M-IMUs in patients with shoulder musculoskeletal disorders still presents open challenges to be overcome in order for them to be used systematically in clinical practice for optimizing patients’ care.

## Data Availability

The datasets used and/or analysed in this study are available from the corresponding author upon reasonable request.
